# Correlation between Bruxism and Gastroesophageal Reflux Disorder and Their Effects on Tooth Wear. A Systematic Review

**DOI:** 10.3390/jcm11041107

**Published:** 2022-02-19

**Authors:** Alessandro Nota, Laura Pittari, Martina Paggi, Silvio Abati, Simona Tecco

**Affiliations:** Dental School, IRCCS San Raffaele Hospital, Vita-Salute San Raffaele University, 20132 Milan, Italy; nota.alessandro@hsr.it (A.N.); martinapaggi98@gmail.com (M.P.); abati.silvio@hsr.it (S.A.); tecco.simona@hsr.it (S.T.)

**Keywords:** sleep bruxism, awake bruxism, gastroesophageal reflux, sleep disorders, enamel wear, review, tooth abrasion

## Abstract

Bruxism and gastroesophageal reflux (GERD) can lead to wear of the dental tissues. Wear has a mechanical or chemical origin, and it is of extrinsic or intrinsic type. Bruxism and GERD are two etiological factors of dental wear. The intrinsic mechanical wear (abfraction) of Bruxism and intrinsic chemical wear (erosion) of GERD are both involved in sleep disorders; indeed, they could have associations and act in synergy in dental wear. The purpose of this review was to find out the possible associations between bruxism and GERD and their effects on tooth wear. The research was conducted on PubMed and the Cochrane Library using the following Keywords/Mesh Terms: Tooth wear, Bruxism, Sleep Bruxism, Sleep Disorders, or GERD. Only systematic reviews and clinical studies performed exclusively on human subjects were included in the review. Initially, the research gave more than 630 results on dental wear, bruxism and GERD and after application of the inclusion criteria irrelevant studies were excluded, and 5 studies were finally included in this review. It was possible to observe the presence of some associations between the two problems (reflux and GERD) and hypothesize negative effects on tooth wear. This research revealed the presence of an interconnection between these three problems (reflux, GERD and tooth wear) that can further act in synergy by attacking the hard dental tissues both from a chemical (reflux) and mechanical (bruxism) point of view. The dentist could play a role of “sentinel” in a multidisciplinary team, intercepting these problems early in order to treat them in the most appropriate way. PROSPERO Registration Number: CRD42021234209.

## 1. Introduction

Bruxism and gastroesophageal reflux (GERD) can lead to wear of the dental tissues [1,2]. Dental wear is a condition with a multifactorial etiology that leads to an irreversible loss of the hard tissues of the tooth, namely enamel, dentin and cement. It can be of mechanical or chemical origin and of an extrinsic or intrinsic type, and bruxism and GERD are two causative factors of dental wear itself [3].

Tooth wear can be divided into two subtypes: mechanical wear (attrition, abfraction and abrasion) and chemical wear (erosion) [4]. According to what is reported in the literature, the prevalence of tooth wear in adults increases from 3% at the age of 20, to 17% at the age of 70, although it has increased sharply in recent years [5,6,7,8].

According to the Montreal definition, GERD is a condition of troublesome symptoms and complications that result from the reflux of stomach contents into the esophagus; This causes bothersome symptoms and/or complications [9,10]. The disease has been sub-classified into esophageal and extra-esophageal syndromes and GERD is recognized as a possible cause of intrinsic chemical wear of the teeth, based on a meta-analysis of Wu et al. and all the past literature [9,11,12]. Diagnosis of GERD is typically based on classic symptoms and response to acid suppression after an empiric trial. GERD is an important health concern as it is associated with decreased quality of life and significant morbidity [13,14].

Regarding bruxism, it is essential to make a distinction between sleep bruxism and awake bruxism. Sleep bruxism is a masticatory muscle activity during sleep characterized as rhythmic (phasic) or non-rhythmic (tonic) movement and is not considered a movement disorder or sleep disorder in otherwise healthy individuals [15,16]. Awake bruxism is an activity of the masticatory muscles during wakefulness that is characterized by repetitive or prolonged contact of the teeth and/or by bracing or thrusting of the mandible and is not considered a movement disorder in otherwise healthy individuals [17,18,19,20]. Bruxism, as a cause of intrinsic mechanical wear (abfraction and attrition), and GERD, as a cause of intrinsic chemical wear (erosion), are both involved in sleep disorders, thus, they could have associations and act in synergy under the profile of dental wear [1,2,17,19,21]. In fact, single wear mechanisms can rarely act alone, but interact with each other [22,23]. Only more recent studies published starting from 2018 began to hypothesize the possible association between GERD and bruxism, but there are only a few studies that address and analyze this possible association [24].

The purpose of this systematic review is to analyze the previous studies regarding the possible associations between tooth wear and sleep disorders, in particular GERD and bruxism, as well as the associations between these disorders themselves.

## 2. Materials and Methods

The review was performed in accordance with PRISMA and registered on the PROSPERO database with registration number CRD42021234209. For the present systematic review of the literature, a search was carried out on the PubMed (US National Library of Medicine), Embase, Scopus and the Cochrane Library databases using the following Keywords/Mesh Terms: Bruxism, Sleep Bruxism, Sleep Disorders, GERD and Tooth wear applying the specific research strategy shown in Table 1. The titles and abstracts (when available) of the publications were reviewed by two operators simultaneously (in case of disagreement a third operator was involved) to determine whether the publications could shed light on the research goal, and only those publications presenting a direct and/or indirect association between tooth wear, reflux and bruxism, bruxism and GERD were selected. Only publications performed exclusively on human subjects dated up to October 2020, were included in the review. The included systematic reviews or studies were in English, Italian, Spanish, French, German and Russian and their full text was available. The publications presenting subjects with rare disorders or syndromes were not included. For each of the selected studies, only the results relating to the correlations between bruxism and GERD and its effects on dental health were considered and reported.

Then, all the data relating to the type and construction of the study (the design, the presence of a control group, the randomization mechanism, the study of the error of the method and the technical description of the methods) were recorded with the aim of evaluating the level of evidence and the quality of the studies that were considered based on these systematic scores that were assigned to the retrieved articles (Table 2), with a maximum score of 10:

- low: total score ≤ 4;
- average: score ≤ 5 and ≤ 9;
- high: >9.

## 3. Results

**Figure 1:**
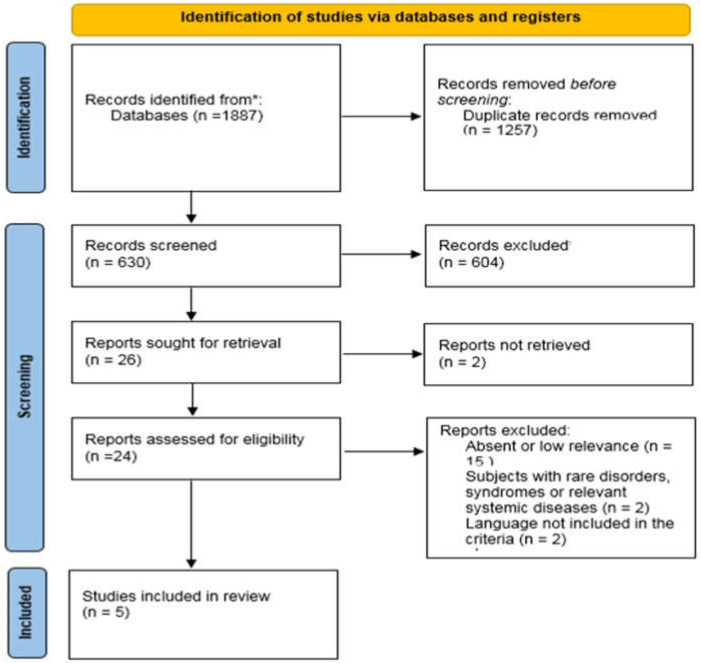
Flow chart.

The search yielded 1887 publications in response to the search strategy used including the keywords; 1882 articles were excluded as not relevant, duplicated, or incompatible with the inclusion criteria. The flowchart is represented in Figure 1.

Following the selection, five publications that observed the presence of some associations between the two problems and hypothesized their negative effects on oral health were included. The qualitative analysis of the studies is summarized in Table 2.

One study was considered of low level, with a score of 4; two studies were considered medium level with scores of 5 and 6, and two studies were considered high with scores of 9 and 10.

The overall level of the studies does not include an adequate number of prospective longitudinal studies that can really clarify the relationship between GERD and bruxism. Table 3 shows and summarizes the results and methods of the articles selected for this review.

The study by Yuanyuan Li et al. [1] demonstrated that GERD is associated with all types of bruxism, with different odds ratios (sleep bruxism with an odds ratio of 6.71; awake bruxism with an odds ratio of 13.06; sleep bruxism and daily bruxism overlap with odds ratio 6.48), with the high OR value for awake bruxism. Furthermore, logistic regression identified an association between the duration of associated GERD and the frequency of bruxism (odds ratio, 1.50). Finally, mediation analysis found that the association between bruxism and GERD was partially mediated by depression, anxiety and reduced sleep quality.

The second study from the same research group [2] confirmed the significant relationship between GERD and bruxism. That study concluded that both conditions are related to tooth wear, with exponential effect, as one condition can aggravate the other, causing more severe tooth wear. Therefore, long-term GERD seems associated with severe tooth wear (mostly, palatal/lingual tooth wear) in patients with bruxism.

Specifically, by analyzing the correlations presented by Li et al. from the studies of July and November 2018 it was possible to obtain a summary table (Table 3), in which all the statistically significant values about the correlation between GERD, bruxism and dental wear are reported.

The other three studies included in the review have lower scientific quality levels than the first mentioned, as they are case-reports and overviews. However, they are considered because they hypothesize the mechanisms of correlations between GERD and bruxism [21,22,24].

Naila Aparecida de Godoi Machado [22] presents a case report in which bruxism is associated with an acidic diet, smoking habit and episodes of gastric reflux, which caused severe tooth wear and great muscular discomfort with daily headache episodes. Therefore, that case-report focused on the multifactorial etiology of dental wear: foreign abrasive substances (e.g., toothbrush and toothpaste); endogenous chemical factors, and exogenous chemical factors from low pH substances, such as citrus fruits. Although some of these dental wear mechanisms can act independently, the authors concluded that there is an association between attritional corrosion (due to bruxism) and the action of a chemical corrosive agent (GERD) in areas where tooth–tooth attrition is given by bruxism. Thus, the study confirmed that these two factors can act in synergy.

Wetselaar et al. [24] wrote a narrative overview describing various sleep disorders, including bruxism and GERD and their association with tooth wear. It was, therefore, hypothesized that GERD can be directly associated with chemical wear of the teeth, while bruxism can be directly associated with mechanical wear, and indirectly also with GERD since both are considered sleep disorders and act on dental wear.

Finally, Lavigne et al. [21] describe bruxism and GERD as conditions related to sleep, which implicated both on tooth wear.

## 4. Discussion

The aim of this study was to review existing scientific literature data on correlations between GERD and bruxism as causes of dental wear and to establish their level of evidence.

GERD is widespread in the western world, its prevalence increases with age and BMI, and men are more frequently affected than women [9,10]. GERD can be considered physiological when it occurs after a meal without further episodes and during pregnancy. It becomes pathological when there is mechanical impairment of the esophagogastric junction and disorders develop [9,10].

Regarding bruxism, a single definition of bruxism is no longer recommended, but two separate definitions are proposed; sleep bruxism which is a masticatory muscle activity during sleep and is not a movement disorder or sleep disorder in otherwise healthy individuals. Awake bruxism is the activity of the masticatory muscles during wakefulness that is characterized by contact repetitive or prolonged dental work and/or by bracing or pushing the mandible, also it is not considered a movement disorder in otherwise healthy individuals [15,17]. Bruxism is considered pathological when a person experiences possible negative consequences, such as dental wear [2,15,23]. The prevalence of bruxism varies from 8 to 31.4%, that of sleep bruxism from 9.7 to 15.9%, depending on the diagnostic methods used.

Dental wear, in addition to being related to GERD, is related to bruxism, considered as a muscular activity. Thus, the analysis of existing literature confirms a probable multifactorial model for tooth wear. Moreover, bruxism and GERD seem to be both risk factors associated with tooth wear [1,2,17,22,24].

Of the five studies analyzed in the present review, two studies have a considerable level of scientific evidence and focus on the relationship of both issues with dental wear. One is a case report focused on bruxism and GERD as coexisting causes of tooth wear. Two other studies are overviews that hypothesize a direct association between GERD and bruxism.

From the study from Lavigne et al., it appeared clear that bruxism and GERD are conditions related to sleep, and both can act by attacking the hard tissues of the tooth. Thus, tooth wear is associated (both directly and indirectly) with sleep disorders [21].

This demonstrates what has already been known for some time, i.e., single wear mechanisms can rarely act alone, but they interact with each other, and an association between bruxism and GERD can also be hypothesized [22].

Therefore, more recent publications of 2018 have paid attention to a possible association between GERD and bruxism on tooth wear [1,2].

The data collected state the existence of an association between GERD and tooth wear, more precisely it can be said that there is an association between GERD and chemical tooth wear and the severity of the wear is related to the severity of the symptoms of GERD. Data also evidence an association between GERD and bruxism. Specifically, as shown in Table 3, in the presence of bruxism, the probability of identifying a concomitant GERD is greater in women than in men, it increases as the duration of GERD increases and it is associated with both awake and sleep bruxism. Thus, it seems that there is an association between GERD and bruxism, mostly awake bruxism. These data are reconfirmed in the November article [2], in which the variable of dental wear is added, showing how at the beginning for a short-term GERD the wear is limited to the lingual surfaces, while after 5 years of presence of the disorder dental wear also extends to other dental surfaces. Thus, long-term GERD seems associated with severe tooth wear (mostly, palatal/lingual tooth wear) in patients with bruxism. Thus, it was suggested that inspection of the oral cavity for intrinsic chemical wear of the teeth should become a routine maneuver in patients with GERD, and cooperation between doctors and dentists is strongly recommended to prevent or improve the possible oral effects of GERD and bruxism synergy.

Both bruxism and GERD fall into the category of sleep disorders, as they occur mainly during the night, both lead to wear of the teeth, and in conclusion, it seems that there is an interconnection between these three problems that are indirectly associated; when the onset of a GERD event precedes the onset of a sleep bruxism event there may be increased tooth wear caused by the demineralization of hard dental tissues by stomach acid which can accelerate the loss of superficial dental tissue through the activity of sleep bruxism.

From a clinical point of view, the results of this literature review can support dental teams. It is essential to identify in the patient who has already been diagnosed with GERD or bruxism, a patient at risk of presenting or developing the other pathology, and therefore, subsequent severe dental wear. An interdisciplinary clinical team will, therefore, be necessary and essential to managing sleep disorders related to oral health. Only a global approach can lead to a correct diagnostic process, and therefore, to optimal assistance. Limits of this review are represented by the small number of publications identified, by the low level of the scientific quality of some of these studies and by the presence of non-homogeneous variables in the various protocols.

The present review is limited to a small number of articles. Although two publications of high scientific evidence were found, both of the publications came from the same scientific group. Thus, the number of studies is limited

## 5. Conclusions

The present systematic review of the existing literature is based on a small number of included articles. With this limit, a probable multifactorial model for tooth wear is confirmed. Bruxism and GERD both seem to be risk factors associated with tooth wear. There is an association between GERD and bruxism, mostly awake bruxism. Long-term GERD seems associated with severe tooth wear (mostly, palatal/lingual tooth wear) in patients with bruxism. Even if the evidence emerged from the considered studies, with clinical relevance, further studies are requested in order to better understand the biological mechanisms behind the described associations.

## Figures and Tables

**Table 1:** Research strategy.

Research Strategy	
1	Bruxism
2	Sleep Bruxism
3	Sleep Disorders
4	Gastroesophageal Reflux
5	Tooth wear
Searches carried out	1. (1 or 2 or 3 or 5) and 4: 811 2. (1 or 2) and (4 or 3 or 5): 1076

**Table 2:** Articles qualitative assessment.

Authors	Article Type	Sample Selection Based on Age Group	Description of an Error Analysis Method	Full Description of the Technical Data	Description of the Blinding Procedure	Preliminary Estimate of the Sample Size	Score
	Systematic review/case control: 3 Other methods: 2 Undefined: 0	Total: 2 Partial: 1 Absent: 0	Yes: 1 No:0	Complete: 2 Partial: 1 Absent: 0	Yes: 1 No: 0	Yes: 1 No: 0	
1. Yuanyuan Li et al.	3	0	2	2	1	1	9
2. Yuanyuan Li et al.	3	2	2	1	1	1	10
3. Naila Aparecida de Godoi Machado et al.	2	2	1	0	0	0	5
4. Peter Wetselaar et al.	3	0	1	1	0	1	6
5. Gilles J. Lavigne et al.	3	0	1	0	0	0	4

**Table 3:** Summary of the results of the studies that analyzed the effects of bruxism and GERD synergy on tooth wear.

Study	Variable 1	Variable 2	Univariate Analysis	Multivariable Analysis (1)	Multivariable Analysis (2)
Yuanyuan Li et al.	Bruxism	GERD	7.95 (5.08–12.46), *p* < 0.001	6.87 (4.34–10.88), *p* < 0.001	
(July 2018)	Bruxism	GERD + Male	4.43 (2.46–7.99), *p* < 0.001	3.81 (2.09–6.95), *p* < 0.001	3.99 (2.17–7.32), *p* < 0.001
	Bruxism	GERD + Female	14.59 (7.02–30.33), *p* < 0.001	12.27 (5.81–25.91), *p* < 0.001	
	Bruxism	Longer GERD duration (>2 years)	1.44 (1.06–1.97), *p*: 0.021	1.50 (1.10–2.05), *p*: 0.011	
	GERD	Awake Bruxism	18.78 (7.91–44.60), *p* < 0.001	13.06 (5.32–32.05), *p* < 0.001	
		Sleep Bruxism	7.61 (4.83–11.98), *p* < 0.001	6.71 (4.22–10.68), *p* < 0.001	
		Awake + Sleep Bruxism	8.14 (3.93–16.87), *p* < 0.001	6.48 (3.05–13.77), *p* < 0.001	
Yuanyuan Li et al.				**OR (95% CI)—*p*-Value**	**OR (95% CI)—*p*-Value**
(November 2018)	Bruxism	GERD	6.21 (3.36–11.46), *p* < 0.001	5.30 (2.62–10.70), *p* < 0.001	3.84 (1.86–7.95), *p* < 0.001
	Bruxism	GERD (duration ≤ 5 years)	4.15 (1.86–9.25), *p* < 0.001	3.38 (1.38–8.24), *p*: 0.008	2.49 (0.98–6.29), *p*: 0.054
	Bruxism	GERD (duration > 5 years)	9.49 (3.70–24.36), *p* < 0.001	8.73 (2.97–25.63), *p* < 0.001	6.27 (2.07–18.97) *p*: 0.001
	Bruxism + GERD ≥ 5 years	Severe Tooth Wear	5.11 (2.60–10.04), *p* < 0.001	4.70 (2.04–10.83), *p* < 0.001	
	Bruxism + GERD ≤ 5 years	Palatal/Lingual Tooth Wear	8.59 (1.94–38.04), *p*: 0.005		
	Bruxism + GERD > 5 years	Palatal/Lingual Tooth Wear	15.19 (4.57–50.51), *p* < 0.001		
	Bruxism + GERD > 5 years	Occlusal/Incisal Tooth Wear	4.86 (2.48–9.52), *p* < 0.001		
	Bruxism + GERD ≤ 5 years	Anterior Severe Tooth Wear	3.10 (1.14–8.45), *p*: 0.027		
	Bruxism + GERD > 5 years	Anterior Severe Tooth Wear	3.16 (1.37–7.32), *p*: 0.007		
		Posterior Severe Tooth Wear	3.92 (1.69–9.05), *p*: 0.001		
		Upper Severe Tooth Wear	3.74 (1.63–8.58), *p*: 0.002		
		Lower Severe Tooth Wear	3.71 (1.60–8.61), *p*: 0.002		

## Data Availability

The data that support the findings of this study are available from the authors, but restrictions to the availability of these data, which were used under license for the current study and so are not publicly available. Data are, however, available from the authors upon reasonable request.
